# Tooth defects of EEC and AEC syndrome caused by heterozygous *TP63* mutations in three Chinese families and genotype‐phenotype correlation analyses of *TP63*‐related disorders

**DOI:** 10.1002/mgg3.704

**Published:** 2019-05-02

**Authors:** Jinglei Zheng, Haochen Liu, Yuan Zhan, Yang Liu, Sing‐Wai Wong, Tao Cai, Hailan Feng, Dong Han

**Affiliations:** ^1^ Department of Prosthodontics, National Engineering Laboratory for Digital and Material Technology of Stomatology, Beijing Key Laboratory of Digital Stomatology Peking University School and Hospital of Stomatology Beijing China; ^2^ National Engineering Laboratory for Digital and Material Technology of Stomatology, Beijing Key Laboratory of Digital Stomatology The 3rd Dental Clinic, Peking University School and Hospital of Stomatology Beijing China; ^3^ Oral and Craniofacial Biomedicine Curriculum, School of Dentistry University of North Carolina at Chapel Hill Chapel Hill North Carolina; ^4^ National Institute of Dental and Craniofacial Research, NIH Bethesda Maryland

**Keywords:** AEC syndrome, EEC syndrome, genotype–phenotype correlation, taurodontism, *TP63*

## Abstract

**Background:**

Ectrodactyly‐Ectodermal dysplasia‐Cleft lip/palate (EEC) syndrome and Ankyloblepharon‐Ectodermal defects‐Cleft lip/palate (AEC) syndrome belong to p63 syndromes, a group of rare disorders exhibiting a wide variety of clinical manifestations. *TP63* mutations have been reported to be associated with both EEC and AEC.

**Methods:**

Analysis of whole exome sequencing (WES) from patients with EEC or AEC syndrome and Sanger sequencing from family members.

**Results:**

We confirmed that three Chinese pedigrees affected with EEC or AEC harboring a distinct *TP63* mutation, and described novel clinical phenotypes of EEC and AEC, including the presence of cubitus valgus deformity and taurodontism, which were discordant to their classical disease features. We also analyzed the genotype–phenotype correlation based on our findings.

**Conclusion:**

We reported that the cubitus valgus deformity in patients with EEC and severe taurodontism in a patient with AEC had not been mentioned previously. Our study expands the phenotypic spectrum of EEC and AEC syndrome.

## 1 INTRODUCTION

The *TP63* gene (OMIM *603273) encodes a member of the p53 family of transcription factors, named tumor protein 63 (p63) (Mills, 2006). Unlike p53, the role of p63 in proliferation, development, and stratified differentiation of epithelial tissues has been widely recognized in human and animal models (Yang et al., 1998). During mouse embryo development, *Tp63* is specifically expressed in the epithelium of multiple organs. Defects of *Tp63^‐/‐^*mice are characterized by ectodermal dysplasia, limb dysplasia, and orofacial deformities, which are consistent with the phenotype of patients carrying a *TP63* mutation (Boughner et al., 2018).

Mutations in the *TP63* gene have been associated with seven diseases: Ectodermal dysplasia–Ectrodactyly Cleft lip/palate syndrome 3 (EEC3, OMIM #604292), Ankyloblepharon‐Ectodermal defects‐Cleft lip/palate syndrome (AEC, OMIM #106260), Rapp‐Hodgkin syndrome (RHS, OMIM 129400), Limb Mammary syndrome (LMS, OMIM #603543), Acro‐Dermato‐Ungual‐Lacrimal‐Tooth syndrome (ADULT, OMIM #103285), Split‐Hand/Foot Malformation type 4 (SHFM4, OMIM #605289) and Isolated cleft lip/cleft palate (orofacial cleft 8, OMIM #129400). These disorders and the localization of their *TP63* mutations reveal a prominent genotype–phenotype correlation (van Bokhoven & Brunner, 2002).

In this study, we detected three heterozygous mutations of *TP63* [c.728G>A (p.R243Q), c.955C>T (p.R319C), c.1769C>T (p.P590L)] in four patients of three Chinese pedigrees identified as having EEC or AEC, and we found some phenotypes which had not been described before. We also summarized the previously reported *TP63* mutations in order to investigate the genotype–phenotype correlation of *TP63*‐related disorders.

## 2 PATIENTS AND METHODS

### 2.1 Pedigrees and patients

Family members of these pedigrees reported to the Department of Prosthodontics, Peking University School of Stomatology (Beijing, China) due to congenital missing teeth. This study was approved by the Ethics Committee of the Peking University School of Stomatology (Beijing, China). All patients provided written informed consent to participate in the study.

### 2.2 DNA sequencing mutation analysis

Genomic DNA from these families was isolated from peripheral blood lymphocytes using a BioTek DNA Whole‐blood Mini Kit (BioTek, Beijing, China). Whole exome sequencing (WES) was conducted by the Beijing Genomic Institute (BGI, China) using DNA from patients to identify potential pathogentic mutations. We filtered all the nonsynonymous single nucleotide polymorphisms (SNPs) (missense, nonsense, and splicing mutations) and inDels (short coding insertions or deletions) based on a minor allele frequency (MAF) ≤0.01, in 1000 Genomes, Exome Aggregation Consortium (ExAC) and Genome Aggregation database. Then according to database of all the genes linked to tooth agenesis which had been published previously, and deleterious result in functional prediction website as Sorting Intolerant from Tolerant (SIFT) and PolyPhen‐2 program, we removed the known variants (present in databases of normal people) as well as the nondeleterious ones. In this way, the number of candidate genes was reduced to two (*TP63*and* BMP4*) in proband #1 and her father; one (*TP63*) in proband #2; three (*TP63, TNNI2,*and* POLA1*) in proband #3. After further bioinformatics and functional prediction analyses of all these three probands and their family members, we excluded other ones but *TP63* (NM_003722). The exonic region of the *TP63* gene of patients and family members was amplified using PCR with Taq PCR Master Mix (BioTek, Beijing, China). The PCR products were sequenced by Tsingke Biological (Beijing, China).

## 3 RESULTS

### 3.1 Clinical findings and mutation detection

#### 3.1.1 Family 1

**Figure 1:**
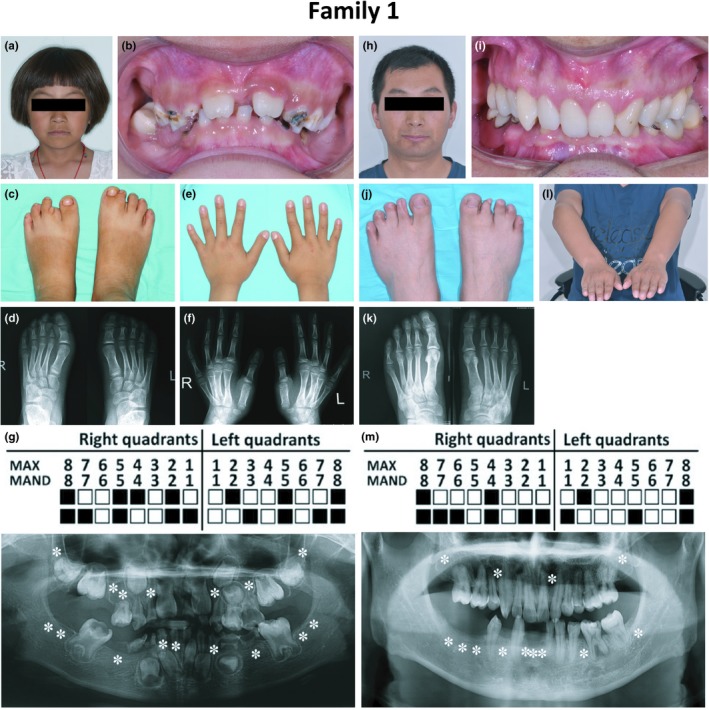
Representative clinical observations from photographic and x‐ray data of Family 1. Proband #1 (a) presented ectodermal phenotype with missing teeth (b) and nail dysplasia (c). Examination revealed cutaneous syndactyly between the first and second toes of the right foot and a missing distal phalanx of the second toe of the left foot (c,d). She had normal hands (e,f). Panoramic radiography showed tooth agenesis and taurodontism of the mandibular first molar (g). Her father (h) presented with tooth agenesis (i, m) and nail dysplasia (j). We found cutaneous syndactyly between the third and fourth toes of both feet and an abnormal second toe on the right foot (j,k). Physical examination revealed cubitus valgus deformity in the right arm (l)

A 6‐year‐old Chinese girl was suspected of having EEC based on clinical manifestations. Physical examination revealed a slight ectodermal phenotype with congenitally missing teeth (Figure 1a,b) and nail dysplasia of the second toe (Figure 1c); her hair was yellow when she was a baby. She exhibited cutaneous syndactyly between the first and second toes of the right foot and was missing the distal phalanx of the second toe of the left foot but had normal hands (Figure 1c–f). Proband #1 also displayed cubitus valgus deformity (picture was not available).

Oral examination showed a congenital lack of deciduous and permanent teeth. Some remaining deciduous teeth in the mouth could be seen with enamel hypoplasia and caries (Figure 1b). Panoramic radiography revealed a congenital absence of multiple permanent teeth, and the bilateral mandibular first molars were taurodont (Figure 1g), which had not been previously reported in conjunction with EEC.

The father of proband #1 had ectodermal dysplasia with tooth agenesis and nail abnormality (Figure 1h–K). He also had cutaneous syndactyly between the third and fourth toes of both feet and malformation of the second toe of the right foot (Figure 1j,k). It is noteworthy that both proband #1 and her father showed cubitus valgus deformities (Figure 1l, the picture of proband #1 was not available), which had not been previously associated with EEC. Her mother did not show any of the phenotypes described above.

The WES and Sanger results revealed that only proband #1 and her father carried a *TP63* mutation c.728G>A (p.R243Q, rs121908836, Figure 4 Family 1), which was first reported by Celli et al. (1999). This mutation is located in exon 5, which encodes the DNA binding domain (DBD). Most mutations in this domain are related to occurrences of EEC. The clinical phenotypes and identification of the mutant locus led to confirmation of EEC syndrome in the proband and her father.

#### 3.1.2 Family 2

**Figure 2:**
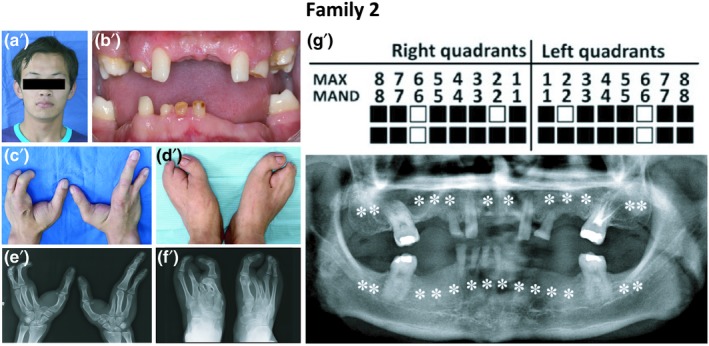
Representative clinical observations from photographic and x‐ray data of Family 2. Proband #2 (a′) presented ectodermal dysplasia including slight sparse and curly hair, tooth agenesis (b′), and dysplastic nails (c′,d′). Both hands and feet were affected by ectrodactyly and cutaneous syndactyly was found between the fourth and fifth toes (c′–f′). Imaging revealed severe tooth agenesis (g′).

An 18‐year‐old Chinese boy was suspected of having EEC based on clinical manifestations and x‐ray image findings. Proband #2 had sparse and curly hair, extensively distributed pigmented nevus on his face and missing teeth (Figure 2a,b). A physical examination revealed ectrodactyly on both hands and feet with missing index and third fingers (toes), and cutaneous syndactyly between the fourth and fifth toes with dysplastic nails (Figure 2c,d). The x‐ray examination showed the missing metacarpal and phalanx of the index finger, the phalanx of the third finger on both hands and the phalanges of the fourth and fifth fingers on the left hand. The phalanges of the second and third toes on both feet and the phalanx of the fourth toe on the left foot were also missing, and the metacarpals of the first to fourth toes were malformed (Figure 2e,f). Sweat function and heat tolerance were reported to be normal. Both of his parents appeared to be normal.

Oral examination and panoramic radiography revealed oligodontia of permanent teeth and spade‐shaped upper lateral incisors. All remaining teeth had intensive carious lesions (Figure 2b,g).

The WES and Sanger sequencing results showed that only proband #2 carried a *TP63* mutation c.955C>T (p.R319C, rs121908839, Figure 4 Family 2), which was first reported by van Bokhoven et al. (2001) (Table S1). This mutation is located in exon 7 of the *TP63* gene and DBD of the protein. The clinical phenotypes and identification of the mutant locus led to the confirmation of EEC Syndrome in proband #2.

#### 3.1.3 Family 3

**Figure 3:**
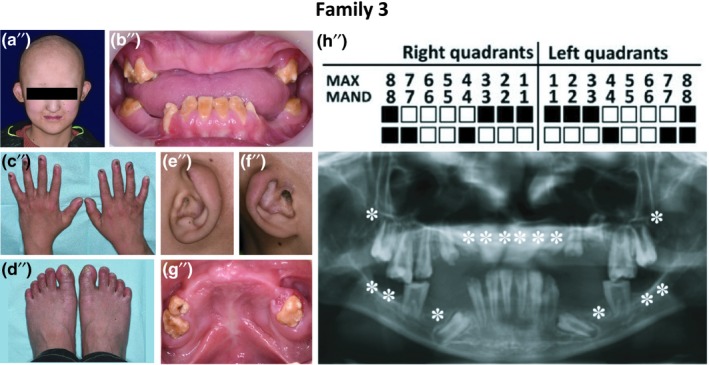
Representative clinical observations from photographic and x‐ray data of Family 3. Family 3: Proband #3 (a′′) presented ectodermal dysplasia, including lack of hair and eyelashes, sparse eyebrows, low functioning sweat glands tooth agenesis (b′′), and nail dysplasia (c′′,d′′). Examination showed nasal stenosis and external auditory canal stenosis of left ear (e′′). The right ear had been surgically restored (f′′). He also suffered from cleft palate, which had been surgically repaired (g′′). Image analysis revealed missing teeth and typical taurodontism of the first molar (h′′)

A 12‐year‐old Chinese boy was suspected of having AEC based on clinical manifestations and x‐ray image findings. A physical examination revealed severe ectodermal phenotypes: lack of hair, sparse eyebrows, no eyelashes, underactive sweat glands, nail dysplasia, and missing teeth (Figure 3a–d). There were many scars on his scalp, due to underdeveloped skin and cutaneous erosions on the scalp at birth. He also suffered from external auditory canal stenosis in both ears (the right had been surgically restored) and nasal stenosis (Figure 3e,f), but his hearing and sense of smell appeared to be normal. Proband #3 was short in stature compared to his peers. No unusual manifestations or mutations were found in his parents.

Oral examination showed multiple congenitally missing permanent teeth. The remaining teeth displayed enamel hypoplasia and dentin exposure (Figure 3b). The left palate had been surgically restored (Figure 3g). Panoramic radiography revealed unclosed apical foramens of premolars and taurodontism of first molars (Figure 3h), which had not been associated with AEC before.

**Figure 4:**
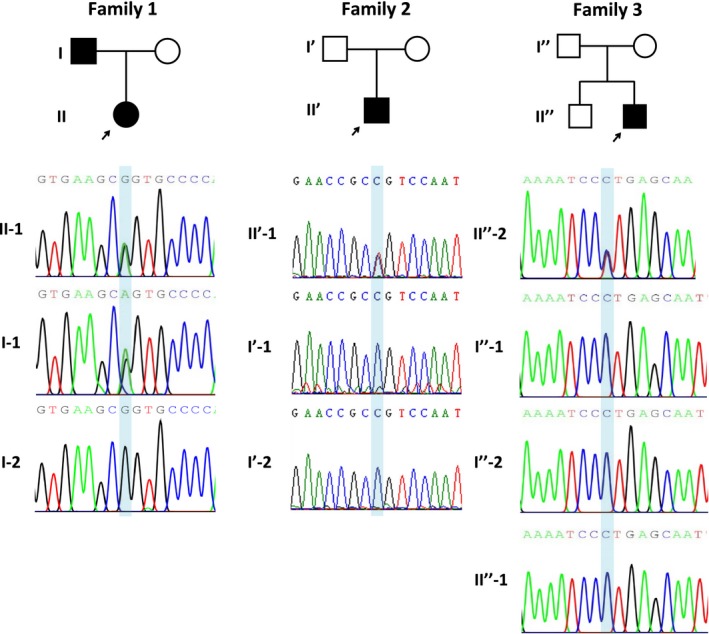
Sequencing chromatogram of three families. Family 1: The proband #1 (II‐1) and her father (I‐1), affected with EEC, were determined to be a heterozygous *TP63* (NM_003722) mutation: c.728G>A (p.R243Q). Family 2: The proband #2 (II′‐1), affected with EEC, was determined to be a heterozygous *TP63* (NM_003722) mutation: c.955C>T (p.R319C). Family 3: The proband #3 (II′′‐2), affected with AEC, was determined to be a heterozygous *TP63* (NM_003722) mutation: c.1769C>T (p.P590L).

The WES and Sanger sequencing results indicated that only proband #3 carried a *TP63* mutation c.1769C>T (p.P590L, CM095572, Figure 4 Family 3), which has been reported by Rinne et al. in 2009 (Rinne, Bolat, Meijer, Scheffer, & van Bokhoven, 2009). This mutation is located in exon 14, which is involved with encoding sterile‐a‐motif domain (SAM). Most of the mutations in this domain are reportedly associated with AEC. The clinical phenotypes and DNA sequencing led to the confirmation of AEC Syndrome in this patient.

### 3.2 Summary of previously reported *TP63* mutations and genotype–phenotype correlation analyses of *TP63*‐related disorders

**Figure 5:**
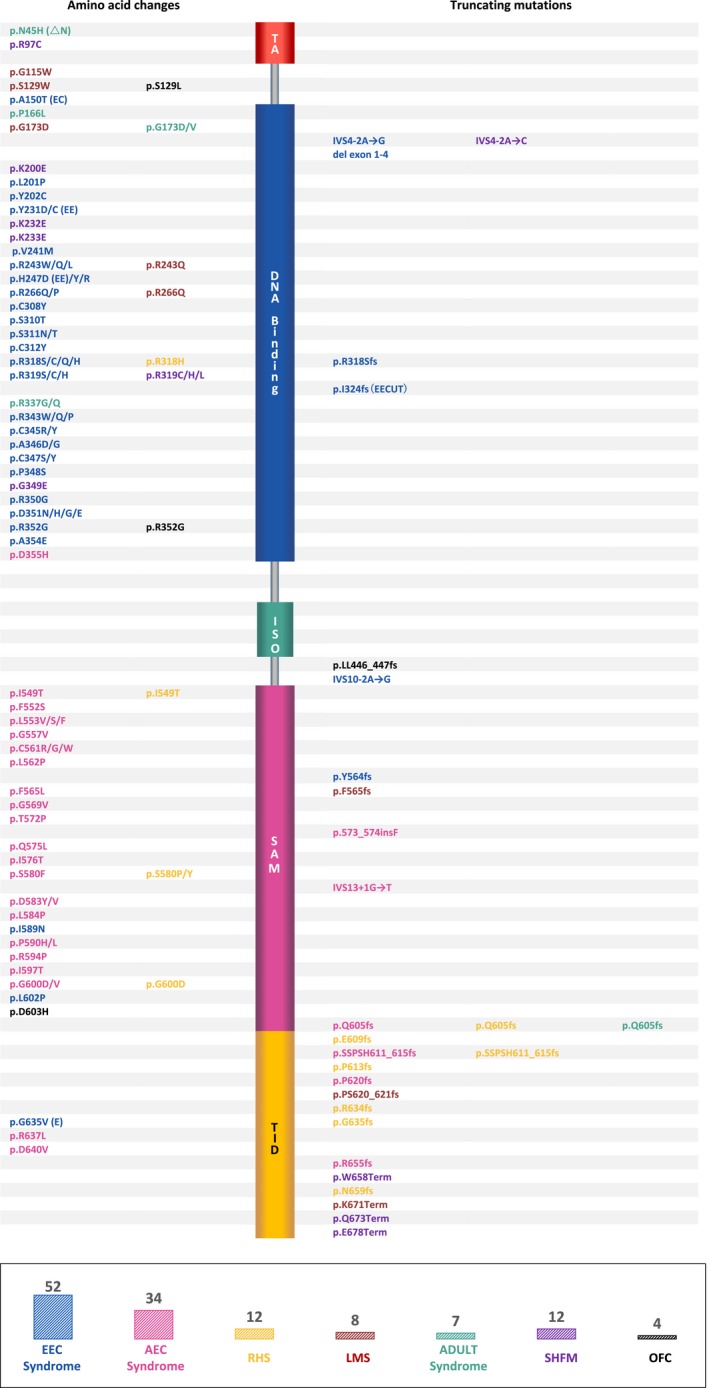
Distribution and number of casual mutations in the *TP63* (NM_003722) gene responsible for ectodermal dysplasia syndromes and disorders, revealing genotype–phenotype correlation (modified according to van Bokhoven & Brunner, 2002)

EEC syndrome is an autosomal dominant disorder characterized by absence of the central parts of the hands and feet, resulting in split‐hand/foot malformation, ectodermal dysplasia, and cleft lip with or without cleft palate (Maas, de Jong, Buss, & Hennekam, 1996) (Table 1). According to the statistics in Human Gene Mutation Database (HGMD) and the ClinVar database, there are 52 different *TP63* mutations associated with EEC (approximately 40.3% of *TP63* mutations), including missense and frameshift mutations (Figure 5). Some individuals show incomplete EEC phenotypes, such as lack of a cleft lip or palate, or have only hand or foot malformations that lead to a cautious differential diagnosis of SHFM. Most of the mutations related to EEC are located in DBD of p63, with the exception of three missense mutations and one frameshift mutation located in another region.

**Table 1:** The summary of phenotypes of *TP63*‐related disorders

Phenotype/Syndrome	Ectodermal dysplasia					Cleft lip/palate	Syndactyly/Ectrodactyly	Ankyloblepharon	Mammary‐gland hypoplasia and/or nipple aplasia	External auditory canal stenosis
	Hypohidrosis	Tooth agenesis	Dry skin	Nail dystrophy	Lacrimal‐duct abnormalities					
EEC3	+++	+++	+++	+++	++	+++	++	–	–	+
AEC	+++	+++	+++	+++	++	+++	±	+++	–	+
RHS	++	++	++	++	+	+	–	–	–	+
LMS	±	±	±	±	+	+	++	–	+++	–
ADULT	–	++	++	++	++	–	++	–	–	–
SHFM4	–	–	–		–	–	+++	–	–	–
OFC8	–	–	–		–	+++	–	–	–	–

Note

+++, characteristic phenotype; ++, regularly observed; +, occasionally observed; ±, seldom observed; –, never observed.

AEC syndrome is also known as “Hay‐Wells syndrome.” Patients with this syndrome show characteristic clinical manifestations, including immature or no limb development, typical facial features and ankyloblepharon, which is rarely seen in other syndromes (Hay & Wells, 1976) (Table 1). However, some AEC patients do not exhibit ankyloblepharon. There are 34 different *TP63* mutations related to AEC (approximately 26.4% of *TP63* mutations), including missense and frameshift mutations (Figure 5). Most AEC‐associated *TP63* mutations are located in SAM domain, with the exception of two missense mutations in DBD and five mutations, including two frameshifts, in transactivation inhibitory domain (TID).

RHS is characterized by the combination of anhidrotic ectodermal dysplasia, cleft lip, and cleft palate (Rapp & Hodgkin, 1968) (Table 1). The clinical features are quite similar to AEC except for the typical facial features and ankyloblepharon. There are 12 different *TP63* mutations associated with RHS (approximately 9.3% of *TP63* mutations), including missense and frameshift mutations (Figure 5). The missense mutations are in DBD. All the frameshifts are in SAM and TID.

The manifestations of LMS are severe hand/foot anomalies and hypoplasia/aplasia of the mammary gland and nipple (van Bokhoven et al., 1999) (Table 1). There are 8 different *TP63* mutations associated with LMS (approximately 6.2% of *TP63* mutations), including missense, nonsense, and frameshift mutations (Figure 5). The missense mutations are located in DBD or are outside of a domain. All frameshift and nonsense mutations are in SAM and TID.

SHFM is characterized as split‐hand/foot malformation (Yang, Lin, Zhu, Luo, & Lin, 2018) (Table 1). There are 12 different SHFM‐related *TP63* mutations (approximately 9.3% of *TP63* mutations), including missense and nonsense mutations (Figure 5). All nonsense mutations are in TID, and the majority of missense mutations is in DBD, except for one in transactivation domain (TA) of the TAp63 isoform. The prevalence of SHFM is low, and most cases might be due to mutations in other genes.

ADULT is associated with ectodermal dysplasia and limb malformations (Azzi et al., 2017) (Table 1). There are 6 different *TP63* missense mutations and one frameshift mutation related to ADULT (approximately 5.4% of *TP63* mutations, Figure 5). Most missense mutations are in DBD, and the frameshift mutation is in SAM.

## 4 DISCUSSIONS

All *TP63* mutations reported previously are heterozygous. Consistent with this, our patients carry heterozygous mutations as well. Therefore, no patients with *TP63* homozygous mutation has been found so far.

Mutations of the *TP63* gene could cause many different syndromic and non‐syndromic diseases (van Bokhoven et al., 2001). The most common manifestations include ectodermal dysplasia, cleft lip and/or palate and limb defects. AEC and RHS share the same phenotypes including ectodermal dysplasia and cleft lip and/or palate, while EEC and LMS patients exhibit all the characteristics. Some individuals show incomplete EEC syndrome phenotypes, such as lack of a cleft lip or palate, or have only hand or foot malformations that lead to a diagnosis of SHFM (van Bokhoven & Brunner, 2002). In our study, genotype–phenotype correlation analyses demonstrated that *TP63* mutations in different domains of p63 could be related to different disorders with complete or incomplete penetrance. However, because of the similarity and overlap of the phenotypes, patients with the same mutation could be diagnosed as different syndromes.

It is noteworthy that tooth defects in patients with *TP63* mutations are quite common. Oligodontia, conoid teeth, enamel hypoplasia, and dentinal dysplasia could result in the physiological dysfunctions and aesthetic defects. In this study, we identified severe taurodontism, which has only been reported once in an RHS patient, in our EEC and AEC patients (proband #1 and proband #3). Moreover, we found cubitus valgus in both proband #1 and her father with *TP63* mutation c.728G>A (p.R243Q). Although split‐hand/foot malformation seems to be quite common in patients with *TP63*‐related disorders, cubitus valgus deformity of arms has not been reported previously. Taken together, our findings broaden the phenotypic spectrum of *TP63*‐related disorders.

## 5 CONCLUSION

In this study, three heterozygous *TP63* mutations were detected in four patients of three Chinese nuclear families identified as having at least one member with EEC or AEC syndrome. For the first time, we reported the cubitus valgus deformity in patients with EEC and severe taurodontism in a patient with AEC. The novel phenotypes found in this study, especially the detailed description of tooth defects, could broaden the phenotypic spectrum of *TP63*‐related disorders.

## CONFLICT OF INTEREST

The authors have no conflict of interest to declare.
