# Supplementary material for: Tooth defects of EEC and AEC syndrome caused by heterozygous TP63 mutations in three Chinese families and genotype‐phenotype correlation analyses of TP63‐related disorders
Source: Mol Genet Genomic Med. 2019 May 2;7(6):e704. doi: 10.1002/mgg3.704 (PMC6565570; doi:10.1002/mgg3.704)
Supplement: Supplementary file 1 [file MGG3-7-e704-s001.docx]

Supplement Table 1 Summary of human TP63 variations reported in HUMD and Pubmed database

| Mutation | | Exon | Domain | Syndrome | No. of patients | Ectodermal dysplasia | Orofacial clefting | Limb | Mammary gland | Ankylobl-epharon | Additional features | Reference |
| --- | --- | --- | --- | --- | --- | --- | --- | --- | --- | --- | --- | --- |
| Nucleotide | Amino Acid |  |  |  |  |  |  |  |  |  |  |  |
|  | p.N45H | 3 | TA2(dN) | **ADULT** | 1 | N, T |  | S | + |  |  | Amiel et al. (2001) |
| c.289C>T | p.R97C | 3 | TA(TA) | **SHFM** | 1 |  |  | E |  |  |  | Zenteno (2005) Am J Med Genet 134A, 74 |
| c.343G>T | p.G115W | 4 |  | **LMS** | 1 |  |  |  |  |  |  | van Bokhoven (2002) Am J Hum Genet 71, 1 |
| c.343G>T | p.G115W | 4 |  | **LMS** | 27 | L, N, S, T | CP |  | + |  | hypohydrosis | van Bokhoven (1999) Am J Med Genet 64, 538 |
| c.386C>G | p.S129W | 4 |  | **LMS** |  |  |  |  |  |  |  | Rinne (2007) Cell Cycle 6, 262 |
| c.386C>T | p.S129L | 4 |  | **NSCL** | 1 |  | CLP |  |  |  |  | Leoyklang (2006) J Med Genet 43, e28 |
| c.448G>A | p.A150T | 4 |  |  | 1 | H, N, S, T | CP |  |  |  | bulbous nose, variegate pigmentation, keratosis pilaris, trichorrhexis nodosa; hypohidrosis | Cabanillas (2011) Pediatr Dermatol 28, 707 |
| c.497C>T | p.P166L | 4 | DBD | **ADULT** | 2 | L, N, S, T |  |  | + |  | fine hair, hypothelia | van Zelst-Stams (2009) Am J Med Genet A 149A, 1558 |
| c.518G>T | p.G173V | 4 | DBD | **ADULT with CP** | 1 | H, L, N, S, T | CP | S | + |  | protruding ears with underdeveloped helices, malar flattening, exfoliative dermatitis, cafe-au-lait spots, and extensive freckling | Prontera (2011) Am J Med Genet A 155, 2746 |
| c.518G>A | p.G173D | 4 | DBD | **LMS/ADULT** | ? | ? |  |  |  |  |  | Rinne (2007) Cell Cycle 6, 263 |
| >191059bp incl ex. 1-4 |  | 1-4 |  | **EEC** | 1 | ? |  |  |  |  |  | Aradhya (2012) Genet Med 14, 594 |
| c.580-2A>G | IVS4-2A→G |  |  | **EEC** | 1 | ? |  |  |  |  |  | Monti (2013) Hum Mutat 34, 894 |
| c.580-2A>C | IVS4-2A→C |  |  | **SHFM** |  |  |  |  |  |  |  | van Bokhoven (2001) Am J Hum Genet 69, 481 |
| c.598A>G | p.K200E | 5 | DBD | **SHFM** | 1 |  | CLP | E |  |  |  | Simonazzi (2012) Prenat Diagn 32, 296 |
| c.602T>C | p.L201P | 5 | DBD | **EEC** | 2 | H, N, T | CLP | E, S |  |  |  | Rinne (2006) Am J Med Genet A 140A, 1396 |
| c.605A>G | p.Y202C | 5 | DBD | **EEC** | 1 | H, L, N, S, T | CP | S | + |  | hypohydrosis | van Bokhoven (2002) Am J Hum Genet 71, 2 |
| c.691T>G | p.Y231D | 5 | DBD | **EEC** |  |  |  |  |  |  |  | Rinne (2007) Cell Cycle 6, 264 |
| c.692A>G | p.Y231C | 5 | DBD | **EE** | 2 | ? |  |  |  |  |  | van Bokhoven (2002) Am J Hum Genet 71, 3 |
| c.694A>G | p.K232E | 5 | DBD | **SHFM** |  |  |  |  |  |  |  | van Bokhoven (2001) Am J Hum Genet 69, 481 |
| c.697A>G | p.K233E | 5 | DBD | **SHFM** | 2 |  |  | E, S |  |  |  | Ianakiev (2000) Am J Hum Genet 67, 59 |
| c.721G>A | p.V241M | 5 | DBD | **EE** | 1 | ? |  |  |  |  |  | van Bokhoven (2002) Am J Hum Genet 71, 4 |
| c.721G>A | p.V241M | 5 | DBD | **EEC** | 1 | H, N, S, T | CLP | E |  |  |  | Pozo (2004) Br J Dermatol 151, 927 |
| c.728G>T | p.R243L | 5 | DBD | **EEC** | 1 | H, L |  | E, S | + |  | anterior hair whorl | de Mollerat (2003) J Med Genet 40, 55 |
| c.727C>T | p.R243W | 5 | DBD | **EEC** | 3 | H, L, N, S, T | CLP | E, S |  |  | renal abnormalities | Celli (1999) Cell 99, 143 |
| c.727C>T | p.R243W | 5 | DBD | **EEC** | 5 | H, L, N, S | CLP | E, S |  |  | mild hypoplastic tibia, vaginal stenosis | van Bokhoven (2001) Am J Hum Genet 69, 481 |
| c.728G>A | p.R243Q | 5 | DBD | **EEC** | 4 | H, T |  | E, S | + |  |  | Celli (1999) Cell 99, 143 |
| c.728G>A | p.R243Q | 5 | DBD | **EEC** | 4 | H, L, N, T | CLP | E, S | + |  | mandibular prognathism | van Bokhoven (2001) Am J Hum Genet 69, 481 |
| c.728G>A | p.R243Q | 5 | DBD | **EEC** | 1 | H, T |  | E, S |  |  |  | de Mollerat (2003) J Med Genet 40, 55 |
| c.728G>A | p.R243Q | 5 | DBD | **EEC** | 1 | + | + | + |  |  |  | Yin (2010) J Dent Res 89, 813 |
| c.728G>A | p.R243Q | 5 | DBD | **EEC** | 1 | L, ? | ? | ? |  |  | eyes abnormalities | Di Iorio (2012) Ophthalmology 119, 74 |
| c.728G>A | p.R243Q | 5 | DBD | **LMS** | 1 | L, N, T |  | S | + |  |  | Rinne (2006) Am J Med Genet A 140A, 1396 |
| c.739C>T | p.H247Y | 5 | DBD | **EEC** | 1 | H, L, N, S, T |  | S |  |  |  | Rinne (2006) Am J Med Genet A 140A, 1396 |
| c.739C>G | p.H247D | 5 | DBD | **EE** | 2 | H, T |  | E, S |  |  | hypohidrosis, hypopigmented | Sorasio (2009) Clin Exp Dermatol 34, e726 |
| c.740A>G | p.H247R | 5 | DBD | **EEC** | 2 | H, N, S, T | CLP | E, S |  |  | eyes and urogenital abnormalities | Clements (2010) Br J Dermatol 162, 201 |
| c.740A>G | p.H247R | 5 | DBD | **EEC** | 2 | L, ? | ? | ? |  |  | eyes abnormalities | Di Iorio (2012) Ophthalmology 119, 74 |
| c.797G>A | p.R266Q | 6 | DBD | **EEC** | 4 | H, L, N, S, T | CP | E | + |  | hypohydrosis, urinary/kidney abnormalities | van Bokhoven (2002) Am J Hum Genet 71, 5 |
| c.797G>A | p.R266Q | 6 | DBD | **EEC** | 22 | H, L, N, S, T | CLP | E, S | + |  | renal abnormalities, micturiction, urethral stenosis, anal stenosis, atrophic bladder epithelium, abnormal inner ear | van Bokhoven (2001) Am J Hum Genet 69, 481 |
| c.797G>A | p.R266Q | 6 | DBD | **LMS** | 3 | H, L, N, S, T |  |  | + |  | urinary/kidney abnormalities, hearing impairment | Rinne (2006) Am J Med Genet A 140A, 1396 |
| c.797G>A | p.R266Q | 6 | DBD | **LMS** | 1 | + |  | + | + |  |  | Yin (2010) J Dent Res 89, 813 |
| c.797G>C | p.R266P | 6 | DBD | **EEC** | 2 | H, S, T | CLP | E, S |  |  | depressed nasal bridge | Sripathomsawat (2011) Am J Med Genet A 155, 228 |
| c.923G>A | p.C308Y | 7 | DBD | **EEC** | 2 | H, L, N | CLP | E, S |  |  |  | van Bokhoven (2001) Am J Hum Genet 69, 481 |
| c.929G>C | p.S310T | 7 | DBD | **EEC** | 1 | Ectodermal dysplasia, T | CLP | E |  |  |  | Yin (2010) J Dent Res 89, 813 |
| c.932G>C | p.S311T | 7 | DBD | **EEC** | 2 | L, ? | ? | ? |  |  | eyes abnormalities | Di Iorio (2012) Ophthalmology 119, 74 |
| c.932G>A | p.S311N | 7 | DBD | **EEC** | 1 | H, L, T | CLP | E, S | + |  |  | Celli (1999) Cell 99, 143 |
| c.932G>A | p.S311N | 7 | DBD | **EEC** | 1 | L, ? | ? | ? |  |  | eyes abnormalities | Di Iorio (2012) Ophthalmology 119, 74 |
| c.935G>A | p.C312Y | 7 | DBD | **EEC** | 1 | H | CLP | E, S |  |  |  | van Bokhoven (2002) Am J Hum Genet 71, 5 |
| c.946A>T | p.M316L | 7 | DBD |  |  |  |  |  |  |  | abnormality of the skeletal system | Retterer (2016) Genet Med 18, 696 |
| c.952C>A | p.R318S | 7 | DBD | **EEC** | 1 | H, N, S, T | CLP | E, S |  |  | eyes and urogenital abnormalities | Clements (2010) Br J Dermatol 162, 201 |
| c.952C>A | p.R318S | 7 | DBD | **EEC** | 1 | L, ? | ? | ? |  |  | eyes abnormalities | Di Iorio (2012) Ophthalmology 119, 74 |
| c.952C>T | p.R318C | 7 | DBD | **EEC** | 4 | H, L, N, S, T | CLP | E, S |  |  | recurrent cholesteatoma | van Bokhoven (2001) Am J Hum Genet 69, 481 |
| c.952C>T | p.R318C | 7 | DBD | **EEC** | 1 | L, ? | ? | ? |  |  | eyes abnormalities | Di Iorio (2012) Ophthalmology 119, 74 |
| c.952C>T | p.R318C | 7 | DBD | **EEC** | 2 | L | CLP | E, S |  |  |  | Dianzani (2003) J Med Genet 40, e133 |
| c.953G>A | p.R318H | 7 | DBD | **EEC** | 4 | H, L, N, S, T | CLP | E, S | + |  | corneal scarring/vision loss, hernia, mental retardation | Celli (1999) Cell 99, 143 |
| c.953G>A | p.R318H | 7 | DBD | **EEC** | 3 | H, L, N, S, T |  | E, S |  |  | mandibular retrognathism, nails on both sides of fifth finger, micturiction, hypohydrosis | van Bokhoven (2001) Am J Hum Genet 69, 481 |
| c.953G>A | p.R318H | 7 | DBD | **EEC** | 8 | ? |  |  |  |  |  | van Bokhoven (2002) Am J Hum Genet 71, 5 |
| c.953G>A | p.R318H | 7 | DBD | **EEC** | 1 | H | CLP | E, S |  |  | hearing loss | de Mollerat (2003) J Med Genet 40, 55 |
| c.953G>A | p.R318H | 7 | DBD | **EEC** | 6 | L, ? | ? | ? |  |  | eyes abnormalities | Di Iorio (2012) Ophthalmology 119, 74 |
| c.953G>A | p.R318H | 7 | DBD | **RHS** | 1 | H, L, N, S, T |  | S |  |  | hypohydrosis, urinary/kidney abnormalities | Bougeard (2003) Eur J Hum Genet 11, 700 |
| c.953-954GC>AA | p.R318Q | 7 | DBD | **EEC** | 1 | H, L, N |  | E, S |  |  | choanal atresia, synblepharon, supernumerary nipple | van Bokhoven (2001) Am J Hum Genet 69, 481 |
| c.953_954delGCinsAA |  | 7 | DBD | **EEC** |  |  |  |  |  |  |  | van Bokhoven (2001) Am J Hum Genet 69, 481 |
| c.955C>A | p.R319S | 7 | DBD | **EEC** | 2 | H, N, S, T |  | E, S |  |  | eczema | van Bokhoven (2001) Am J Hum Genet 69, 481 |
| c.955C>T | p.R319C | 7 | DBD | **EEC** | 15 | H, L, N, S, T | CLP | E, S |  |  | photophobia, mandibular retrognathism | van Bokhoven (2001) Am J Hum Genet 69, 481 |
| c.955C>T | p.R319C | 7 | DBD | **EEC** | 5 | ? |  |  |  |  |  | van Bokhoven (2002) Am J Hum Genet 71, 7 |
| c.955C>T | p.R319C | 7 | DBD | **EEC** | 1 | L, ? | ? | ? |  |  | eyes abnormalities | Di Iorio (2012) Ophthalmology 119, 74 |
| c.955C>T | p.R319C | 7 | DBD | **SHFM** | 2 |  |  | E, S |  |  |  | Ianakiev (2000) Am J Hum Genet 67, 59 |
| c.955C>T | p.R319C | 7 | DBD | **EEC** | 3 | H, L, N, S, T | CLP | E, S | + |  |  | Barrow (2002) J Med Genet 39, 559 |
| c.955C>T | p.R319C | 7 | DBD | **EEC** | 8 | H, S, T | CLP | E, S |  |  |  | Ray (2004) Clin Genet 66, 217 |
| c.956G>A | p.R319H | 7 | DBD | **EEC/SHFM** | 1 | H, L, S, T |  | E, S |  |  | eczema | van Bokhoven (2001) Am J Hum Genet 69, 481 |
| c.956G>T | p.R319L | 7 | DBD | **SHFM** | ? |  |  |  |  |  |  | Carter (2017) J Hum Genet 62, 877 |
| c.970_972delATT |  | 7 | DBD | **EECUT** |  | H, L, N | CP | E, S |  |  | urinary tract abnormalities and thymic abnormalities, small ear canals, kidney abnormalities | Giampietro (2013) Am J Med Genet A 161, 1432 |
| c.1009C>G | p.R337G | 8 | DBD | **ADULT** |  |  |  |  |  |  |  | Rinne (2006) Am J Med Genet A 140A, 1396 |
| c.1010G>A | p.R337Q | 8 | DBD | **ADULT** | 8 |  |  |  |  |  |  | Duijf (2002) Hum Mol Genet 11, 799 |
| c.1010G>A | p.R337Q | 8 | DBD | **ADULT** | 2 | ? |  |  |  |  |  | van Bokhoven (2002) Am J Hum Genet 71, 7 |
| c.1027C>T | p.R343W | 8 | DBD | **EEC** | 2 | H, L, N, S, T | CLP | S |  |  | renal abnormalities, hearing loss | Celli (1999) Cell 99, 143 |
| c.1027C>T | p.R343W | 8 | DBD | **EEC** | 1 | H, L, N | CLP | S, E |  |  |  | van Bokhoven (2001) Am J Hum Genet 69, 481 |
| c.1027C>T | p.R343W | 8 | DBD | **EEC** | 2 | ? |  |  |  |  |  | van Bokhoven (2002) Am J Hum Genet 71, 7 |
| c.1027C>T | p.R343W | 8 | DBD | **EEC** | 1 | H, N | CLP | S |  |  |  | de Mollerat (2003) J Med Genet 40, 55 |
| c.1027C>T | p.R343W | 8 | DBD | **EEC** | 1 | L, ? | ? | ? |  |  | eyes abnormalities | Di Iorio (2012) Ophthalmology 119, 74 |
| c.1028G>A | p.R343Q | 8 | DBD | **EEC** | ? | H | CP |  | + |  |  | Ianakiev (2000) Am J Hum Genet 67, 61 |
| c.1028G>A | p.R343Q | 8 | DBD | **EEC** | 5 | H, L, N, S, T | CLP | S, E |  |  | renal abnormalities, photophobia, uterine fibroma | van Bokhoven (2001) Am J Hum Genet 69, 481 |
| c.1028G>A | p.R343Q | 8 | DBD | **EEC** | 2 | H, L, N, T | CLP | Cleft |  |  |  | de Mollerat (2003) J Med Genet 40, 55 |
| c.1028G>A | p.R343Q | 8 | DBD | **EEC** | 6 | L, ? | ? | ? |  |  | eyes abnormalities | Di Iorio (2012) Ophthalmology 119, 74 |
| c.1028G>A | p.R343Q | 8 | DBD | **EEC** | 5 | H, L, N, S | CLP | polydactyly, E, S |  |  |  | Dianzani (2003) J Med Genet 40, e133 |
| c.1028G>C | p.R343P | 8 | DBD | **EEC** | 1 | H, T | CL | E, S |  |  | blindness, seizures | de Mollerat (2003) J Med Genet 40, 55 |
| c.1033T>C | p.C345R | 8 | DBD | **EEC** | 1 | N, S, T |  | E, S |  |  |  | Celli (1999) Cell 99, 143 |
| c.1034G>A | p.C345Y | 8 | DBD | **EEC** | 1 | N, T | CLP | E, S |  |  | hypopigmentation, hypoplastic brows and lashes | Lehmann (2005) Eur J Pediatr 164, 530 |
| c.1037C>A | p.A346D | 8 | DBD | **EEC** | 2 | H, N, S, T | CLP | E, S |  |  |  | Clements (2010) Br J Dermatol 162, 202 |
| c.1037C>G | p.A346G | 8 | DBD | **EEC** | 3 | H, L, T | CLP | E, S |  |  | dracryocystitis with corneal ulceration, blepharitis, blepharophimosis, bilateral sensorineural hearing loss, hypothyroidism | Alves (2015) Genet Mol Biol 38, 37 |
| c.1039T>A | p.C347S | 8 | DBD | **EEC** | 1 | H, L, N, S, T |  | S |  |  | high palate, hypertelorism, small eyes, recurrent chest infections, eczema | van Bokhoven (2001) Am J Hum Genet 69, 481 |
| c.1040G>A | p.C347Y | 8 | DBD | **AEC** | 1 | H |  | E |  |  | diffuse erosions, erythema, desquamation; hypoplastic alae nasi, and a short philtrum; post-axial polydactyly; anal atresia with rectovestibular fistula; atrial septal defect and patent ductus arteriosus | Celik (2011) Am J Med Genet A 155, 3100 |
| c.1042C>T | p.P348S | 8 | DBD | **EEC** | 1 | H, L, N, S, T |  | E, S |  |  |  | van Bokhoven (2001) Am J Hum Genet 69, 481 |
| c.1046G>A | p.G349E | 8 | DBD | **SHFM** | 4 |  |  | E, S |  |  |  | Luo (2008) Mutat Res 637, 182 |
| c.1048A>G | p.R350G | 8 | DBD | **EEC** | 1 | H, N, S, T | CLP | E |  |  | eyes abnormalities | Clements (2010) Br J Dermatol 162, 201 |
| c.1048A>G | p.R350G | 8 | DBD | **EEC** | 1 | L, ? | ? | ? |  |  | eyes abnormalities | Di Iorio (2012) Ophthalmology 119, 74 |
| c.1051G>A | p.D351N | 8 | DBD | **EEC** |  |  |  |  |  |  |  | Rinne (2006) Am J Med Genet A 140A, 1396 |
| c.1051G>C | p.D351H | 8 | DBD | **EEC** | 1 | H, L, N, S, T | CLP | E, S |  |  | unilateral hydrocele, photophobia, chronic blepharitis, corneal ulcers | van Bokhoven (2001) Am J Hum Genet 69, 481 |
| c.1052A>G | p.D351G | 8 | DBD | **EEC** | 1 | H, L, N, S, T | CLP | E, S |  |  | hypopigmented skin; absent eyebrows; flat and low nasal bridge, short philtrum; maxillary hypoplasia; narrow auditory canals, mild hearing loss and almost blind; no secondary sexual characteristics | Akahoshi (2003) Am J Med Genet A 120A, 370 |
| c.1053C>A | p.D351E | 8 | DBD | **EEC** | 1 | H, N, S, T |  | E |  |  |  | Clements (2010) Br J Dermatol 162, 201 |
| c.1054A>G | p.R352G | 8 | DBD | **EEC/NSCL** | ? | ? |  |  |  |  |  | Rinne (2007) Cell Cycle 6, 265 |
| c.1054A>G | p.R352G | 8 | DBD | **NSCL** | 1 |  | CLP |  |  |  |  | Leoyklang (2006) J Med Genet 43, e29 |
| c.1061C>A | p.A354E | 8 | DBD | **EEC** |  |  |  |  |  |  |  | Rinne (2007) Cell Cycle 6, 265 |
| c.1063G>C | p.D355H | 8 | DBD | **AEC** | 1 | L, N, S | CP |  |  | + | erythrodermia and skin erosions in the scalp | Gonzalez (2017) Ophthalmic Genet 38, 277 |
| c.1338_1341delACTT | p.LL446_447fs | 10 |  | **NSCL** | 3 |  | CLP |  |  |  | larygeal cleft | Wenger (2018) Am J Med Genet A 176, 75 |
| c.1350-2A>G | IVS10-2A→G |  |  | **EEC** | ? |  |  |  |  |  |  | Barrow (2002) J Med Genet 39, 559 |
| c.1646T>C | p.I549T | 12 | SAM | **AEC** | 1 | H, L, N, S, T |  | S |  | + | scalp dermatitis, peculiar facies | Bertola (2004) Clin Genet 66, 79 |
| c.1646T>C | p.I549T | 12 | SAM | **RHS** | 1 | H, L, N, S | CP | S |  |  | erythematous lesion in the scalp, back and genitalia, characteristic facies | Bertola (2004) Clin Genet 66, 79 |
| c.1655T>C | p.F552S | 13 | SAM | **AEC** | 1 | N | CLP | E, S |  | + | erythroderma, erosions, microcephaly, alopecia, absent eyebrows and eyelashes | Berk (2009) Pediatr Dermatol 26, 617 |
| c.1657T>G | p.L553V | 13 | SAM | **AEC** | 1 | H, L, N, S, | CLP |  |  | + | cutaneous erosions, absent eyebrows and eyelashes, mid-face hypoplasia, ventral hypospadias, blepharitis and keratopathy, conductive hearing loss | McGrath (2001) Hum Mol Genet 10, 221 |
| c.1658T>C | p.L553S | 13 | SAM | **AEC** | 1 | H, L, N, S | CLP | S | + | + | skin erosions, absent eyebrows and eyelashes, hyponychial, auditory canals abnormal, | Payne (2005) Arch Dermatol 141, 1567 |
| c.1659A>T | p.L553F | 13 | SAM | **AEC** | 1 | H, L, N, S, | CLP |  |  | + | cutaneous erosions, absent eyebrows and eyelashes, mid-face hypoplasia, ventral hypospadias, blepharitis and keratopathy, external auditory hypoplasia, hearing impairment | McGrath (2001) Hum Mol Genet 10, 221 |
| c.1670G>T | p.G557V | 13 | SAM | **AEC** |  |  |  |  |  |  |  | Rinne (2007) Cell Cycle 6, 266 |
| c.1681T>G | p.C561G | 13 | SAM | **AEC** | 1 | S | CLP |  | + | + | cutaneous erosions , hypospadias, aplastic external auditory meati | McGrath (2001) Hum Mol Genet 10, 221 |
| c.1681T>C | p.C561R | 13 | SAM | **AEC** | 1 | H, N, S | CP | E |  | + | cutaneous erosions, stenotic ear canals, conductive hearing loss | Chiu (2011) Pediatr Dermatol 28, 15 |
| c.1683T>G | p.C561W | 13 | SAM | **AEC** | 1 | H, S, T | CLP | S |  | + | cutaneous erosions, mid-face hypoplasia, corneal scarring, hearing impariment | McGrath (2001) Hum Mol Genet 10, 221 |
| c.1685T>C | p.L562P | 13 | SAM | **AEC** | 2 | H, N | CP | E, S |  |  | cutaneous erosions, mandibular hypoplasia, ectropion | Barbaro (2012) Am J Med Genet A 158A, 1957 |
| c.1689_1690insA |  | 13 | SAM | **EEC** | 1 | L, T | CLP | E, S | + |  | hearing impariment | Celli (1999) Cell 99, 143 |
| c.1693_1694delTT |  | 13 | SAM | **LMS** | 1 | T | CP | E, S | + |  |  | van Bokhoven (2001) Am J Hum Genet 69, 481 |
| c.1695C>A | p.F565L | 13 | SAM | **AEC** | 2 | ? |  |  |  |  |  | Rinne (2009) Am J Med Genet A 149A, 1948 |
| c.1706G>T | p.G569V | 13 | SAM | **AEC** | 1 | H, S, T | CLP | S |  | + | cutaneous erosions, mid-face hypoplasia, corneal scarring, hearing impariment | McGrath (2001) Hum Mol Genet 10, 221 |
| c.1714A>C | p.T572P | 13 | SAM | **AEC** | 2 | H, L, N, T | CP | S | + | + | alopecia, absence of one parotid gland and duct | McGrath (2001) Hum Mol Genet 10, 221 |
| c.1718_1720dupTTC | p.573_574insF | 13 | SAM | **AEC** | 1 | H, N, S | CLP |  |  |  | auricle deformity (low-set, posterior rotated, narrowing), skin erosions, stenotic auditory canals | Tsutsui (2003) Br J Dermatol 149, 395 |
| c.1724A>T | p.Q575L | 13 | SAM | **AEC** | 2 | H, N, T | CP |  |  |  | blepharitis | McGrath (2001) Hum Mol Genet 10, 221 |
| c.1727T>C | p.I576T | 13 | SAM | **AEC** | 1 | H, L, N, S, | CLP |  |  | + | cutaneous erosions, absent eyebrows and eyelashes, mid-face hypoplasia, ventral hypospadias, blepharitis and keratopathy, conductive hearing loss | McGrath (2001) Hum Mol Genet 10, 221 |
| c.1738T>C | p. S580P | 13 | SAM | **RHS** | 1 | H, L, N, S, T | CLP |  | + |  | taurodontism, external auditory canal stenosis, hearing impairment, hypoplastic scapulae | Kantaputra (2003) J Dent Res 82, 433 |
| c.1739C>A | p. S580Y | 13 | SAM | **RHS** | 1 | H, L, N, S, T | CLP |  |  |  |  | Shotelersuk (2005) Clin Exp Dermatol 30, 282 |
| c.1739C>T | p.S580F | 13 | SAM | **AEC** | ? |  |  |  |  |  |  | Bertola (2004) Clin Genet 66, 80 |
| c.1747G>T | IVS13+1G→T | 14 | SAM | **AEC** | 1 | ? |  |  |  |  |  | Rinne (2009) Am J Med Genet A 149A, 1948 |
| c.1747G>T | p.D583Y | 14 | SAM | **AEC** | 1 | ? |  |  |  |  |  | Beaudry (2009) Am J Med Genet A 149A, 1952 |
| c.1748A>T | p.D583V | 14 | SAM | **AEC** | 1 | ? |  |  |  |  |  | Rinne (2009) Am J Med Genet A 149A, 1948 |
| c.1751T>C | p.L584P | 14 | SAM | **AEC** | 1 | ? |  |  |  |  |  | Rinne (2009) Am J Med Genet A 149A, 1948 |
| c.1766T>A | p.I589N | 14 | SAM | **EEC** | 1 | ? |  |  |  |  |  | Al-Gazali (2010) Hum Mutat 31, 505 |
| c.1769C>A | p.P590H | 14 | SAM | **AEC** |  |  |  |  |  |  |  | Hida (2014) Eur J Dermatol 24, 495 |
| c.1769C>T | p.P590L | 14 | SAM | **AEC** | 1 | ? |  |  |  |  |  | Rinne (2009) Am J Med Genet A 149A, 1948 |
| c.1781G>C | p.R594P | 14 | SAM | **AEC** | 1 | N, S | CP |  | + | + | erosive scalp dermatitis, hyponychia, webbed penis, skin erosions | Payne (2005) Arch Dermatol 141, 1568 |
| c.1790T>C | p.I597T | 14 | SAM | **AEC** | 1 | H, N | CP |  |  |  | hypoplastic mandible, cutaneous erosions | Sawardekar (2011) Pediatr Dermatol 28, 313 ; |
| c.1799G>T | p.G600V | 14 | SAM | **AEC** | 2 | ? |  |  |  |  |  | Beaudry (2009) Am J Med Genet A 149A, 1953 |
| c.1799G>T | p.G600V | 14 | SAM | **AEC** | 2 | ? |  |  |  |  |  | Rinne (2009) Am J Med Genet A 149A, 1948 |
| c.1799G>A | p.G600D | 14 | SAM | **AEC** | 3 | ? |  |  |  |  |  | Rinne (2009) Am J Med Genet A 149A, 1948 |
| c.1799G>A | p. G600D | 14 | SAM | **RHS** | 3 | H, L, N, S, T | CLP |  |  | + | midfacial hypoplasia, ear abnormalities, hearing impairment, kidney/GU abnormalities | Steele (2005) Pediatr Dermatol 22, 415 |
| c.1805T>C | p.L602P | 14 | SAM | **EEC** | 1 | H, N, T |  | S |  |  |  | Rinne (2006) Am J Med Genet A 140A, 1396 |
| c.1807G>C | p.D603H | 14 | SAM | **NSCL** | 1(+1normal dad has) |  | CLP |  |  |  |  | Leoyklang (2006) J Med Genet 43, e30 |
| c.1815delG | p.Q605fsX94 | 14 | SAM | **RHS/AEC/ADULT** | 1 | H, L, N, T | CP |  |  |  | skin erosion, freckling | Serra (2011) Am J Med Genet A 155, 3104 |
| c.1827delA | p.E609fs | 14 | TID | **RHS** | 1 | H, N, T | CP |  |  |  | hypohydrosis, genitourinary(kidney/GU) abnormalities | Bougeard (2003) Eur J Hum Genet 11, 700 |
| c.1833_1843dupCTCCCCTTCTC | p.SSPSH611_615fs | 14 | TID | **AEC / RHS** |  |  |  |  |  |  |  | Prontera (2008) Genet Couns 19, 397 |
| c.1838delC | p.P613fs | 14 | TID | **RHS** | 2 | H, N, S, T | CP |  | + |  | narrow pinched nose, small chin | Kannu (2006) Am J Med Genet A 140A, 888 |
| c.1838delC | p.P613fs | 14 | TID | **RHS** | 1 | H, N, T |  |  |  |  | hypohydrosis | Rinne (2006) Am J Med Genet A 140A, 1396 |
| c.1859delC | p.P620fs | 14 | TID | **AEC** | 1 | H, L, N, S, T | CLP |  |  |  | hypohydrosis, hearing impariment | van Bokhoven (2002) Am J Hum Genet 71, 7 |
| c.1860_1861delAA | p.PS620_621fs | 14 | TID | **LMS** | 1 | L, N, S | CP | E,S | + |  | anteriorly placed anus, earpits | van Bokhoven (2001) Am J Hum Genet 69, 481 |
| c.1900delC | p.R634fsX70 | 14 | TID | **RHS** | 2 | H, S, T |  |  | + |  | mild deafness, corneal dystrophy | Holder-Espinasse (2007) Eur J Hum Genet 15, 1115 |
| c.1904G>T | p.G635V | 14 | TID | **Ectodermal dysplasia** | 11 | H, N, S, T |  |  |  |  | lip pits, plantar keratoderma | Goldsmith (2014) J Invest Dermatol 134, 2277 |
| c.1904delG | p.G635fsX68 | 14 | TID | **RHS** | 1 | H, L, S, T | CP |  |  |  | mid-facial hypoplasia and a narrow nose, hearing impairment | Chan (2005) Clin Exp Dermatol 30, 183 |
| c.1910G>T | p.R637L | 14 | TID | **AEC** | 5 | ? |  |  |  |  |  | Rinne (2009) Am J Med Genet A 149A, 1948 |
| c.1919A>T | p.D640V | 14 | TID | **AEC** | 1 | ? |  |  |  |  |  | Rinne (2009) Am J Med Genet A 149A, 1948 |
| c.1963delC | p.R655fsX665 | 14 | TID | **AEC** | 3 | ? |  |  |  |  |  | Rinne (2009) Am J Med Genet A 149A, 1948 |
| c.1974G>A | p.W658Term | 14 | TID | **SHFM** | 1 |  |  | E, S |  |  |  | Sowińska-Seidler (2014) J Appl Genet 55, 105 |
| c.1976delA | p.N659fsX44 | 14 | TID | **RHS** | 3 | H, L, N, T | CLP |  |  | + | anhidrosis, typical facial features: narrow nostrils, maxillary hypoplasia, small mouth, thin lips, small teeth with enamel defects and missing teeth, reduced production of tears and saliva; pigmented naevi | Dianzani (2003) J Med Genet 40, e133 |
| c.2011A>T | p.K671Term | 14 | TID | **LMS** | 1 | L, N, T | CLP | E, S | + |  | hypohydrosis | Rinne (2006) Am J Med Genet A 140A, 1396 |
| c.2017C>T | p.Q673Term | 14 | TID | **SHFM** |  |  |  |  |  |  |  | van Bokhoven (2001) Am J Hum Genet 69, 481 |
| c.2032G>T | p.E678Term | 14 | TID | **SHFM** | 1 |  |  |  |  |  |  | van Bokhoven (2002) Am J Hum Genet 71, 6 |
| c.+374 G > A |  |  |  |  |  |  |  |  |  |  | unicornuate uterus, polycystic ovary syndrome, Müllerian duct anomalies? | Wang (2016) Hum Reprod 31, 2865 |
| c.+2345 C > T |  |  |  |  |  |  |  |  |  |  | associated with a decreased risk for bladder cancer | Wang (2016) Int J Cancer 139, 65 |
| c.-6957 C > T |  |  |  |  |  |  |  |  |  |  | association with susceptibility to Lung adenocarcinoma | Hosgood (2012) Hum Genet 131, 1197 |
| c.-33879 C > T |  |  |  |  |  |  |  |  |  |  | association with susceptibility to Lung adenocarcinoma | Miki (2010) Nat Genet 42, 893 |

Note:

1  Ectodermal dysplasia: H = sparse hair; L = lacrimal-duct abnormalities (alacrimia); N = nail dystrophy; S = dry skin (hypohidrosis); T = tooth agenesis;

2 Orofacial clefting: CL = cleft lip; CP = cleft palate;

3 Limb: E = ectrodactyly; S = syndactyly;

4  Mammary-gland hypoplasia and/or nipple aplasia.

5  patients who have been classified as having EEC syndrome have strong phenotypic overlap with LMS. The same as AEC syndrome and LMS.

6 dN: △Np63, one of the p63 isoform encoded from promoter 2; TA: TAp63, one of the p63 isoform produced by activating promoter 1.

7 Three families reported in this article highlight by red color.

8 The version number for *TP63* is NM_003722.
